# Machine Learning-Based Near-Infrared Laser Leakage Detection System for Wine Bottles

**DOI:** 10.3390/s26144474

**Published:** 2026-07-14

**Authors:** Xinyu Chen, Jingwen Tan, Shugui Ding, Xiaojun Jin, Ying Jiang

**Affiliations:** 1 College of Mechanical and Electronic Engineering, Nanjing Forestry University, Nanjing 210037, China; 2 Lehui International Institute of Intelligent Packaging, Nanjing Forestry University, Nanjing 211102, China; 3 National Engineering Research Center of Biomaterials, Nanjing Forestry University, Nanjing 210037, China

**Keywords:** near-infrared laser, leakage detection, ethanol concentration, wine bottle packaging

## Abstract

Traditional methods for wine bottle packaging leakage detection often suffer from low efficiency, high false-positive rates, or an inability to detect micro-leakages. This paper proposes a near-infrared laser leakage detection system based on tunable diode laser absorption spectroscopy at 1392 nm, combined with a LightGBM machine learning model. The system detects gaseous ethanol vapor escaping from leaking bottles, addressing the spectral interference caused by ambient water vapor. A total of 1410 samples were collected, and each raw 2000-point spectral contour was compressed into a 200-dimensional feature vector through baseline correction, Z-score normalization, and uniform down-sampling. A two-stage hyperparameter optimization strategy yielded the optimal LightGBM configuration with a 5-fold cross-validation. For the binary classification task, the model achieved an AUC of 0.9949 and an inference speed of 0.0058 ms per sample on a CPU, outperforming Random Forest, PLS, and four deep learning models. For the regression task, the model achieved an R^2^ of 0.5854 ± 0.0919. An anti-interference experiment on 422 samples under varying flow rates, temperatures, and commercial wine types confirmed the model’s robustness, achieving an overall accuracy of 0.94 and an alcohol recall of 0.99. To further validate the system under realistic conditions, a simulated micro-leakage test was conducted using a negative-pressure extraction method: 320 samples were collected from artificially damaged commercial wine bottles placed in a custom-built acrylic vacuum chamber that replicates the production line enclosure. The model achieved an accuracy of 0.95 with zero false negatives. The complete detection cycle takes no more than 5 s per bottle, enabling non-destructive, rapid, and online packaging integrity assessment. The results demonstrate that the proposed system provides a low-cost and reliable solution for wine bottle leakage detection suitable for industrial deployment.

## 1. Introduction

Wine bottle leakage, occurring at either the mouth or body due to sealing failures or material defects, severely impacts product quality, shelf-life stability, and brand reputation [1,2,3]. Even minor leakage can lead to the volatilization of aroma components, while severe leakage contaminates packaging and triggers customer returns [4,5]. Therefore, developing rapid, non-destructive, and reliable leakage detection systems for high-speed production lines is of great significance for minimizing economic losses and ensuring product standardization [6,7].

Wine bottle leakage detection methods primarily rely on visual inspection, weight checking, or acoustic testing, which often suffer from low efficiency, high false-positive rates, or an inability to detect micro-leakages. Near-infrared (NIR) laser spectroscopy offers a promising alternative by targeting the characteristic absorption of ethanol [8]. However, the majority of existing NIR-based studies have primarily focused on the quantitative analysis of liquid ethanol, such as monitoring ethanol concentration in fermentation processes or analyzing molecular interactions in liquid ethanol-water mixtures. While these methods achieve high accuracy in controlled liquid environments, they face significant challenges when applied to leakage detection, where the actual target is gaseous ethanol (ethanol vapor) escaping from the bottle [9,10].

Detecting gaseous ethanol presents unique difficulties compared to liquid analysis [11]. The concentration of leaked ethanol vapor is typically much lower, highly dispersed, and highly susceptible to environmental interference [12,13]. A critical limitation in practical NIR gaseous leakage detection is the strong spectral overlap and interference from ambient water vapor, particularly when using single-wavelength measurements (e.g., at 1392 nm) [14,15,16]. Traditional linear models struggle to maintain accuracy under such fluctuating background conditions [17]. Although machine learning (ML) has demonstrated powerful feature recognition capabilities in other inspection tasks [18,19,20,21], its potential to resolve spectral interference in NIR-based gaseous leakage detection remains underutilized [22].

Furthermore, the proposed approach aims to capture and process the non-linear features embedded in the spectra. Specifically, these nonlinearities primarily arise from three aspects [23,24]: (1) severe spectral overlapping between the absorption bands of ethanol and ambient water vapor; (2) environmental interferences, such as temperature and pressure fluctuations, which cause baseline drift and absorption line broadening; and (3) deviations from the ideal Beer–Lambert law in complex detection environments. While traditional linear models (e.g., PLS) often struggle to effectively decouple these intertwined effects, machine learning (ML) algorithms can fully exploit the redundant information from the target wavelength and its adjacent bands to construct nonlinear, robust prediction models that effectively mitigate water vapor interference in gas-phase measurements.

To address these challenges, this paper proposes a Machine Learning-based Near-Infrared Laser Leakage Detection System for wine bottles. At the hardware level, the system employs Tunable Diode Laser Absorption Spectroscopy (TDLAS). This technique utilizes a highly monochromatic semiconductor laser source whose emission wavelength can be finely tuned by modulating its injection current and temperature. This allows for precise scanning across the characteristic absorption lines of ethanol vapor near 1392 nm, providing high-fidelity spectral data based on the Beer–Lambert law [25]. Unlike traditional single-wavelength linear methods designed for liquids, our approach leverages ML algorithms to process the multi-band NIR spectral data of ethanol vapor, effectively filtering out environmental interference and enhancing detection robustness [26,27]. The main contributions of this study are summarized as follows:

(1) We propose a novel NIR leakage detection framework specifically tailored for gaseous ethanol, integrating adjacent-band spectral features with machine learning to overcome the water vapor interference inherent in single-wavelength (1392 nm) gas-phase measurements.
(2) We develop and compare multiple nonlinear ML prediction models, identifying the optimal algorithm that balances detection accuracy and computational efficiency for real-time production lines.
(3) We validate the proposed system through comprehensive experiments on actual wine bottles, demonstrating its superior robustness and reliability compared to traditional linear chemometric methods.

## 2. Design Approach

### 2.1. Laser Selection

**Figure 1:**
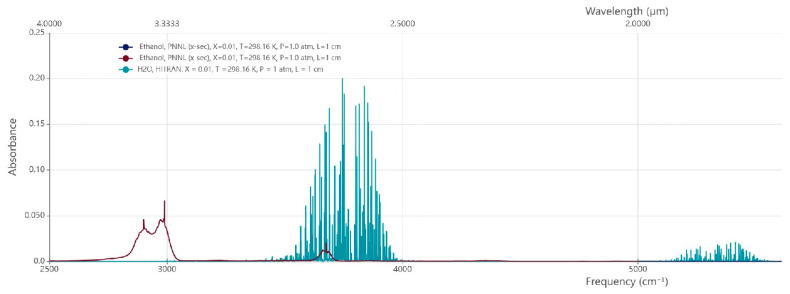
Absorption spectrum of alcohol from 1333 nm to 2000 nm.

As shown in Figure 1, the absorption line characteristics of ethanol vapor in this study were referenced from the HITRAN database [11], and spectral simulations were performed using the SpectraPlot online tool. The calculations were conducted with an optical path length of 1 cm, an ambient pressure of 1 atm, and a temperature of 298.1 K. Combined with the absorption cross-section data of ethanol, a simulated absorbance spectrum was obtained for a specific wavelength range.

The selection of the target wavelength is fundamentally guided by the molecular vibrational transitions of ethanol. The characteristic absorption peak of ethanol vapor at 1392 nm (approx. 7184 cm^−1^) is primarily attributed to the first overtone of the O-H stretching vibration (2ν O-H), with minor contributions from the second overtone and combination bands of C-H stretching vibrations [8,9,11].

We specifically selected the 1392 nm O-H first overtone band because it exhibits a relatively high molar absorptivity compared to other near-infrared bands, making it highly sensitive for detecting the low-concentration ethanol vapor escaping from micro-leaks. However, as correctly governed by its O-H origin, this band is inherently susceptible to severe spectral overlap and interference from ambient water vapor (H_2_O), which also possesses strong O-H absorption features in the 1350–1450 nm region [14]. Traditional single-wavelength linear models struggle to decouple the ethanol signal from the fluctuating water vapor background at this specific band. Furthermore, while traditional multi-wavelength differential techniques are often employed to suppress such interference, they require strictly matched reference wavelengths and extremely high optical path stability; otherwise, new errors may be introduced [15,16].

To overcome these limitations, our detection strategy integrates physical evaporation kinetics with advanced data-driven algorithms. Physically, due to the well-known differences in volatility between ethanol (boiling point ~78.4 °C) and water (boiling point 100 °C), ethanol evaporates and diffuses much more rapidly in the initial moments of a leak. Therefore, the core problem statement of this study focuses on capturing the transient leakage signal during the early stages of a leak. In this initial period, a large quantity of alcohol evaporates rapidly, creating a distinct ethanol-rich concentration spike before the slower-evaporating water vapor accumulates to levels that would severely complicate the spectral analysis.

Algorithmically, rather than avoiding this interference-prone band, our proposed approach leverages machine learning algorithms to process multi-band spectral data around 1392 nm. By exploiting the subtle nonlinear differences in the absorption profiles of ethanol vapor and water vapor across adjacent wavelengths, the ML model effectively mitigates cross-sensitivity. This allows the system to fully exploit the redundant information from the target wavelength and its adjacent bands, constructing a nonlinear and robust prediction model that ensures highly reliable and rapid leak detection.

### 2.2. LightGBM Machine Learning Algorithm

LightGBM (Light Gradient Boosting Machine) [23] is an efficient implementation of the Gradient Boosting Decision Tree (GBDT) framework. Compared with conventional GBDT algorithms, LightGBM significantly improves training speed and reduces memory consumption while maintaining comparable accuracy. One of its core innovations is the introduction of the histogram algorithm [28,29].

In conventional pre-sorting methods, the algorithm needs to compute the sorted positions of all samples for each feature, resulting in time complexity proportional to the product of the number of samples and the number of features. In contrast, the histogram algorithm discretizes continuous floating-point feature values into k discrete integer bins, with each bin recording the cumulative sum of gradients and the number of samples.

Consequently, when searching for the best split point, only at most k bins need to be traversed, greatly reducing the complexity. Since k is typically much smaller than the number of samples, and memory storage is compressed from 64-bit floating point to 8-bit integer, memory usage can be reduced to one-eighth of that of traditional methods. During node splitting in the decision tree, LightGBM adopts a gain formula based on the first-order derivative g_i_ and second-order derivative h_i_. Let the current node sample set be O, the left child node sample set be L, the right child node sample set be R, and the regularization coefficient be λ. The split gain Δ is then:

(1) $$\Delta =\;\frac{{\left(\sum_{i\in L}g_{i}\right)}^{2}}{\sum_{i\in L}h_{i}+\lambda }\;+\;\frac{{\left(\sum_{i\in R}g_{i}\right)}^{2}}{\sum_{i\in R}h_{i}+\lambda }-\frac{{\left(\sum_{i\in o}g_{i}\right)}^{2}}{\sum_{i\in o}h_{i}+\lambda }$$

This metric quantifies the reduction in the loss function before and after splitting; a larger Δ indicates a more optimal split point. The algorithm traverses all possible split points across all features and selects the feature and threshold that maximize Δ for splitting.

For near-infrared spectral data, where the number of spectral channels often amounts to hundreds or thousands, the histogram algorithm effectively compresses the information of each band, enabling the model to run smoothly on ordinary computers.

Another important innovation of LightGBM is Gradient-based One-Side Sampling (GOSS) and Exclusive Feature Bundling (EFB) [30]. GOSS selects samples based on the absolute value of the gradient: samples with large gradients contain more information gain and are therefore fully retained; samples with small gradients are randomly sampled at a certain proportion, and a weight factor is applied to the sampled samples when computing the gain to compensate for the distribution shift. Specifically, the top a% of samples with the largest gradients are retained (without sampling), and from the remaining samples with smaller gradients, b% are randomly selected. The weight factor is:

(2) $$\tilde{g}\;=\;\frac{1\;-\;a}{b}\;\cdot g$$

This strategy can substantially reduce the number of samples involved in each iteration without significantly altering the data distribution, thereby accelerating training.

EFB (Exclusive Feature Bundling) is used to handle high-dimensional sparse features: mutually exclusive features are bundled into a single feature. For example, multiple features generated by one-hot encoding can be losslessly merged, reducing the feature dimension without sacrificing accuracy. In addition, LightGBM adopts a leaf-wise growth strategy with depth limitations. At each step, it selects the leaf node with the maximum gain from all current leaf nodes to split, as opposed to the level-wise growth strategy used in conventional algorithms. Under the same number of leaf nodes, the leaf-wise strategy achieves lower training loss. Let the current set of leaf nodes be L, and let the optimal split gain corresponding to each leaf node k be Δ_k_. The selected leaf node is then:

(3) selected_leaf = arg Δk k∈Lmax 

The objective of this study is to achieve high-precision prediction of alcohol concentration in wine bottles based on near-infrared spectral data in the band around 1392 nm, combined with machine learning algorithms, for the ultimate purpose of online monitoring of wine bottle packaging leakage. LightGBM offers several unique advantages in this scenario. First, it can efficiently handle high-dimensional, small-sample data. Near-infrared spectral data typically contain hundreds of wavelength channels, while the number of samples collected in practical experiments is relatively limited, representing a typical high-dimensional small-sample problem.

The EFB mechanism of LightGBM can automatically perform correlation analysis and bundling for dimensionality reduction on spectral bands, while the histogram algorithm greatly reduces memory consumption. This allows model training to be completed on ordinary hardware without relying on expensive high-performance computing equipment. Second, LightGBM exhibits strong nonlinear modeling capability, effectively addressing water vapor interference.

As previously noted, the region around 1392 nm exhibits both the characteristic absorption of the C–H bond in ethanol and strong interference from the rotational-vibrational absorption lines of water vapor in the 1390–1420 nm range. Traditional linear models, such as partial least squares (PLS), are unable to effectively separate the contributions of alcohol from those of water in the mixed signal. In contrast, LightGBM, as an ensemble tree model, can automatically capture the complex nonlinear relationship between alcohol concentration and spectral signals through the aggregation of multiple decision trees. The GOSS mechanism further selects key samples carrying high information content, enabling the model to focus on subtle differences in spectral morphology between the alcohol signal and the interfering signal. By incorporating information from adjacent bands, it automatically compensates for nonlinear drift caused by fluctuations in ambient humidity, thereby maintaining prediction stability in complex environments.

While providing prediction accuracy superior to that of traditional linear models, LightGBM offers significantly faster training speed than deep neural networks. In the context of online detection of wine bottle packaging leakage, the model may require periodic retraining or fine-tuning in response to environmental changes (e.g., temperature, humidity, bottle type). The high iterative efficiency of LightGBM substantially reduces model maintenance costs.

Based on the above advantages, LightGBM is highly suitable as the core modeling algorithm for this project, providing reliable technical support for rapid, accurate, and non-destructive detection of alcohol concentration related to wine bottle leakage.

## 3. Experiments and Results

### 3.1. Experimental System

**Figure 2:**
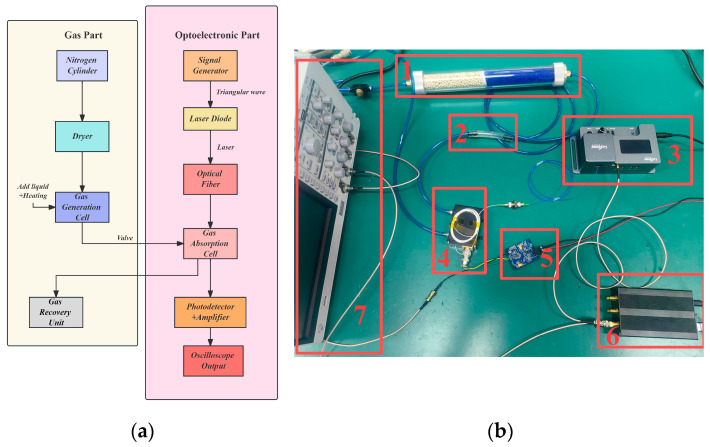
Experimental workflow diagram: (**a**) Conceptual diagram of the experimental workflow; (**b**) Experimental setup. Numbers 1–7 indicate the dryer, gas generation cell, laser diode, gas absorption cell, photodetector with amplifier, signal generator, and oscilloscope.

The experimental setup is illustrated in Figure 2a. In the optoelectronic subsystem, a signal generator outputs a triangular wave signal to drive a laser source, which emits a laser beam. The beam is guided through an optical fiber into a gas absorption cell, where it interacts sufficiently with the gas inside. The transmitted light then undergoes photoelectric conversion via a photodetector and an amplification circuit, and the resulting electrical signal is finally recorded by an oscilloscope.

In the gas handling subsystem, a nitrogen cylinder is connected via rubber tubing. The nitrogen passes through a drying tube to eliminate moisture interference before entering a gas generation cell. The liquid analyte can be introduced into this cell, where heating is applied to accelerate evaporation for the preparation of ethanol and water vapors. By adjusting the valves, the nitrogen carrier gas slowly pushes the target gas into the absorption cell for laser absorption, after which the exhaust gas is discharged into a gas recovery system.

**Figure 3:**
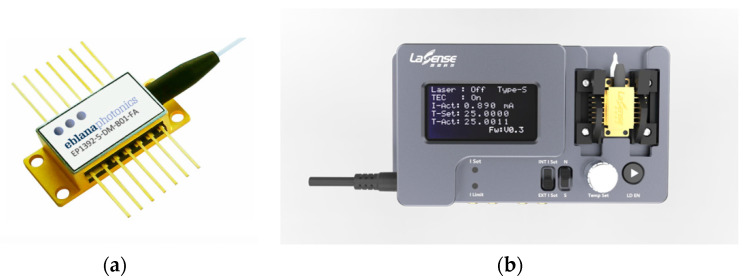
Laser source: (**a**) EP1392-DM-B semiconductor laser; (**b**) M01 laser driver.

As shown in Figure 3, the EP1392-DM-B laser diode (Eblana Photonics) is a semiconductor laser based on an InP-based strained quantum-well structure with a discrete mode (DM) ridge waveguide design. It operates in continuous-wave (CW) mode and is coupled to a single-mode fiber (9/125 μm core/cladding diameter). The output power in the fiber is 5 to 12 mW (typical: 8 mW). The beam is guided through the optical fiber directly into the gas absorption cell; therefore, the spot size at the measurement point is determined by the fiber core diameter and the collimation optics integrated into the gas cell.

The laser wavelength was controlled by modulating the injection current using the M01 laser driver, sourced from Eblana Photonics Ltd. (headquartered in Dublin, Ireland). The driver generated a triangular scanning voltage from 850 mV to 1500 mV (with 0 mV initial offset) at a period of 50 ms, which modulated the injection current within the range of approximately 80 mA to 120 mA. The current tuning coefficient is 8–10 pm/mA, and the temperature tuning coefficient is 0.07–0.1 nm/°C. An integrated thermoelectric cooler (TEC) and thermistor feedback maintained the case temperature at 25 °C. According to the manufacturer’s datasheet, the laser has a threshold current of 15–20 mA, a side-mode suppression ratio (SMSR) of 30–40 dB, and an optical linewidth of <2 MHz. It is packaged in a hermetically sealed 14-pin butterfly package with an integrated optical isolator.

**Figure 4:**
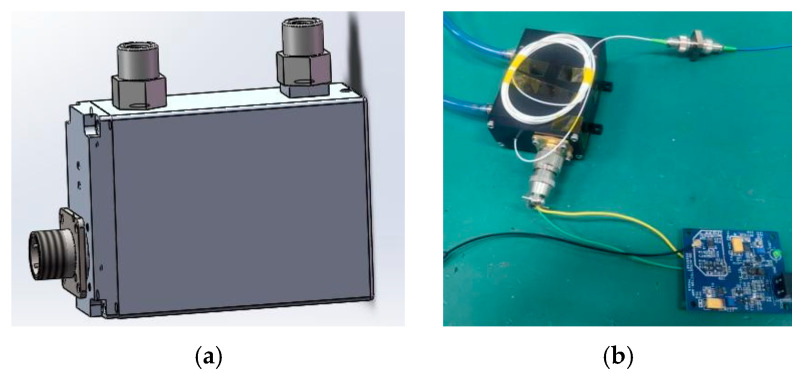
Schematic diagram of the gas absorption cell; (**a**) Schematic diagram of the gas absorption cell; (**b**) Photograph of the gas absorption cell.

In the gas absorption section, as shown in Figure 4, a gas absorption cell with a 3 m optical path length and an optical-input/electrical-output configuration is employed. This cell adopts a proprietary flat-wave optical path structure and incorporates high-stability optical packaging technology. Its core components comprise the cavity, mirrors, standard fiber-optic interfaces, gas flow channels, and a vibration-isolating base.

Benefiting from its unique suspended optical path design, the device exhibits exceptional resistance to vibration and temperature drift. Specifically, this suspended structure mechanically decouples the critical optical components, such as the laser source, collimator, and detector, from the external housing, effectively isolating them from high-frequency mechanical shocks and vibrations encountered in industrial environments. Furthermore, the suspension design minimizes thermal stress and structural deformation caused by ambient temperature fluctuations, thereby maintaining precise optical alignment without the need for frequent recalibration. Combined with active temperature stabilization of the laser source and low-noise signal processing techniques, the system ensures long-term stable operation under complex operating conditions. This makes it particularly well-suited for online real-time gas monitoring applications. Additionally, the system features inherent low-noise characteristics, fully meeting the stringent requirements for trace gas analysis.

In addition to the core instruments mentioned above, the experiment also employed an oscilloscope, a preamplifier, and a Mini dual-channel lock-in amplifier (based on FPGA + ARM architecture). The M01 laser driver was configured to generate a triangular scanning voltage from 850 mV to 1500 mV with a 0 mV initial offset at a period of 50 ms, which modulates the injection current to sweep the laser wavelength across the 1392 nm absorption line. The photodetector signal was preamplified and then fed into the lock-in amplifier for fundamental (1f) demodulation, with the reference frequency set to the modulation frequency, a time constant of 50 ms, and a 24 dB/oct low-pass filter. The demodulated 1f output was recorded by the oscilloscope and used for subsequent feature extraction.

### 3.2. Evaluation Metrics

Mean Absolute Error [31,32]: MAE represents the mean of the absolute errors between the predicted and ground-truth values, reflecting the magnitude of the average deviation in the model’s predictions. The formula is expressed as follows:

(4) $$\mathrm{MAE}\;=\frac{1}{n}\sum_{i=1}^{n}\left|y_{i}\;-\tilde{y_{i}}\right|$$

Root Mean Square Error [33]: RMSE is the square root of the mean of the squared prediction errors, which imposes a heavier penalty on larger errors. Its formula is given as follows:

(5) $$\mathrm{RMSE}\;=\sqrt{\frac{1}{n}\sum_{i=1}^{n}{\left|y_{i}-\tilde{y_{i}}\right|}^{2}}$$

RMSE is highly sensitive to outliers and increases significantly in the presence of large errors. Typically, RMSE ≥ MAE; a larger discrepancy between the two indicates the presence of a small number of samples with substantial errors.

Coefficient of Determination [33]: R^2^ quantifies the extent to which the model explains the variability of the observed data, representing the percentage of the variance in true mean values that can be accounted for by the predictions.

(6) $$R^{2}=1-\frac{\sum_{i=1}^{n}{\left(y_{i}-\tilde{y}\right)}^{2}}{\sum_{i=1}^{n}{\left(y_{i}-{\bar{y}}_{i}\right)}^{2}}$$

where $\tilde{y}$ denotes the mean of true mean values.

Where $y_{i}$ denotes the true value, $\tilde{y_{i}}$ denotes the predicted value, and ${\bar{y}}_{i}$ denotes the mean of the true values.

Characteristics: R^2^ typically ranges from 0 to 1, with values closer to 1 indicating a better model fit and the ability to explain most of the variance in the data. A negative R^2^ indicates that the model’s predictive performance is worse than simply predicting the mean.

### 3.3. Ethanol Feature Extraction Experiment

In practical production scenarios, when leakage occurs from wine bottles, the ethanol vapor produced by the evaporation of the leaked liquid often coexists with water vapor. Water vapor also exhibits dense rovibrational absorption lines in the near-infrared region, which spectrally overlap with the characteristic absorption of the C–H bonds in ethanol, thereby significantly interfering with the accurate measurement of ethanol concentration. Therefore, extracting the spectral features of ethanol under clean background conditions is crucial for subsequent model training [11,14,16,17].

In near-infrared spectroscopic measurements, ambient air contains not only water vapor but also components such as carbon dioxide and oxygen, which exhibit characteristic absorption and introduce additional background noise. Therefore, this experiment employs nitrogen as the background gas to effectively eliminate the spectral contributions of other atmospheric components. In theoretical analysis, nitrogen, as a homonuclear diatomic molecule, exhibits no infrared activity in the near-infrared region and is therefore expected to cause no absorption interference with the target laser. The nitrogen gas used in this study was industrial-grade high-purity nitrogen with a certified purity of 99.99%. The carrier gas (N2) flow rate was set to 200 mL/min using a calibrated mass flow controller (MFC) sourced from MKS Instruments Inc. (Andover, MA, USA) with an accuracy of ±1% of full scale. The gas absorption cell was operated at ambient pressure (101.325 kPa), as its outlet was open to the atmosphere. Unless otherwise stated, all spectra shown in this paper represent voltage signals acquired after photoelectric conversion, which are electrically equivalent to the optical absorption spectra.

**Figure 5:**
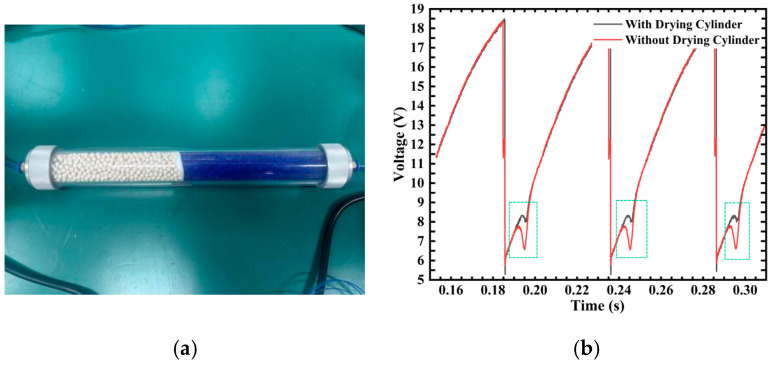
Nitrogen absorption experiment: (**a**) Nitrogen drying apparatus; (**b**) Comparison before and after nitrogen drying.

However, as shown by the white curve in Figure 5b, which displays the absorption profile of the nitrogen before drying, a certain degree of laser attenuation is still observed when the nitrogen is introduced (as highlighted in the red box). This absorption feature closely resembles the spectral lines of water vapor, suggesting that trace amounts of moisture were introduced into the industrial-grade nitrogen due to environmental factors or cylinder impurities. To ensure the absolute absence of water vapor impurities and obtain a truly clean baseline that solely reflects the intrinsic absorption of the ethanol sample, the nitrogen stream was further passed through a desiccant drying tube filled with molecular sieves before entering the gas generation and absorption cells. This rigorous purification step completely eliminates trace moisture, preventing any unintended spectral overlap during the baseline calibration and background subtraction processes, thereby significantly enhancing measurement accuracy.

To address this, a drying apparatus, as illustrated in Figure 5a, was incorporated into the optical path for nitrogen pre-treatment. The blue curve in Figure 5b depicts the absorption profile of nitrogen after passing through the drying canister. As indicated in the green box, the attenuation of the absorption peak for the yellow curve is reduced compared to that of the red curve.

It is important to clarify that the drying process employed in this study relies on the physical adsorption mechanism of molecular sieves, rather than a chemical reaction. Molecular sieves are crystalline aluminosilicates with uniform microporous structures. Due to the strong polarity and suitable kinetic diameter of water molecules, they are selectively trapped within the micropores via van der Waals forces and electrostatic interactions, while the non-polar nitrogen molecules pass through unimpeded. This physical adsorption process does not involve the breaking or formation of chemical bonds, ensuring the chemical integrity of the background gas. By physically removing the moisture, the spectral interference in the 1390–1420 nm region is effectively eliminated, providing a reliable baseline for subsequent ethanol feature extraction.

This result demonstrates a significant decrease in absorbance after drying, confirming that the interference originated from moisture mixed in the industrial nitrogen rather than from other substances. Although a trace amount of moisture remains after drying, its absorbance was reduced to a level comparable to the noise floor of the measurement system, corresponding to approximately 1–2% of the ethanol absorbance signal. At this level, the residual water vapor has no significant impact on subsequent experiments. Therefore, the dried nitrogen is deemed suitable for use as the background gas in this experiment, as it provides a stable and reproducible baseline spectrum with no detectable water vapor features after background subtraction.

Transmittance calibration was performed using a reference-based method. Before each measurement session, a background spectrum was recorded by passing the laser beam through the gas absorption cell filled with dry nitrogen (after passing through the molecular sieve drying tube). This background signal, denoted as I_0_, represents the incident light intensity without any ethanol absorption. During sample measurement, the transmitted light intensity through the gas cell containing the ethanol vapor sample was recorded as I. The transmittance T was then calculated as the ratio T = I/I_0_, implemented through a point-by-point division of the two contours using MATLAB software (R2026a, MathWorks, Natick, MA, USA). This method effectively cancels out wavelength-dependent variations in laser output power, the spectral response of the photodetector, and optical losses in the beam path. Additionally, a dark-current measurement (with the laser turned off) was subtracted from both the background and sample signals to eliminate the detector offset and electronic noise. The entire calibration process was automatically performed by MATLAB scripts before each measurement to ensure consistency.

**Figure 6:**
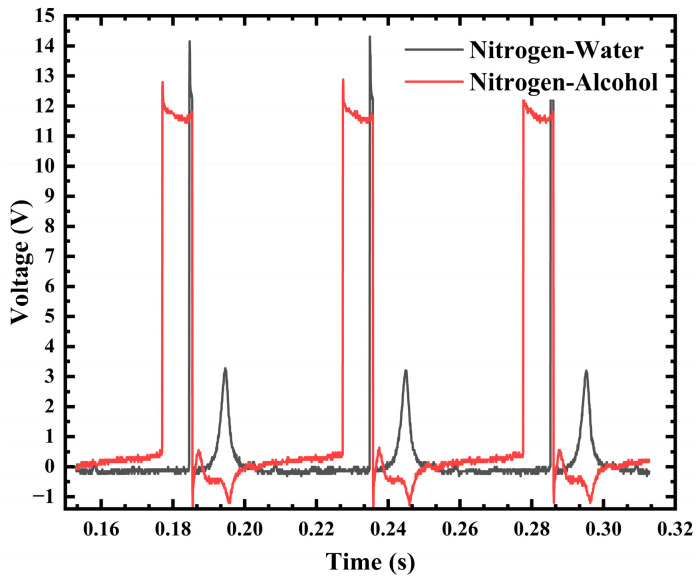
Schematic diagram of the differential spectra of ethanol and water vapor.

To compare the differences in the characteristic absorption spectra of water vapor and ethanol against the nitrogen background, this study employs differential absorption spectroscopy [34]. Specifically, the nitrogen background spectrum is subtracted from the raw absorption spectra of the ethanol and water vapor samples to obtain their respective differential absorption spectra, with the results presented in Figure 6. This differential processing effectively eliminates interference from background baseline drift and environmental noise, highlighting the absorption features of the target components near 1392 nm. Consequently, it intuitively reveals the differences in spectral profiles and absorption intensities between water vapor and ethanol.

As can be seen from Figure 6, following the differential processing for the ethanol-nitrogen and water vapor-nitrogen systems, all curves exhibit the typical periodically repeating features of wavelength modulation differential absorption. The overall absorbance of the nitrogen-ethanol system is significantly higher than that of the nitrogen-water vapor system. The nitrogen-ethanol system exhibits broader absorption peaks, with uniform light intensity attenuation throughout the modulation period. In contrast, the light intensity attenuation in the nitrogen-water vapor system is almost entirely concentrated at the absorption peaks. The differential spectra clearly reveal sharper peak profiles, with virtually no attenuation outside the absorption peaks. Repeated experiments were conducted by varying the injected volumes of ethanol and water vapor.

Furthermore, to investigate the effect of ethanol concentration on the spectral response characteristics and to provide reference data with concentration gradients for the subsequent development of a regression model, a multi-gradient concentration experiment was designed in this study.

**Figure 7:**
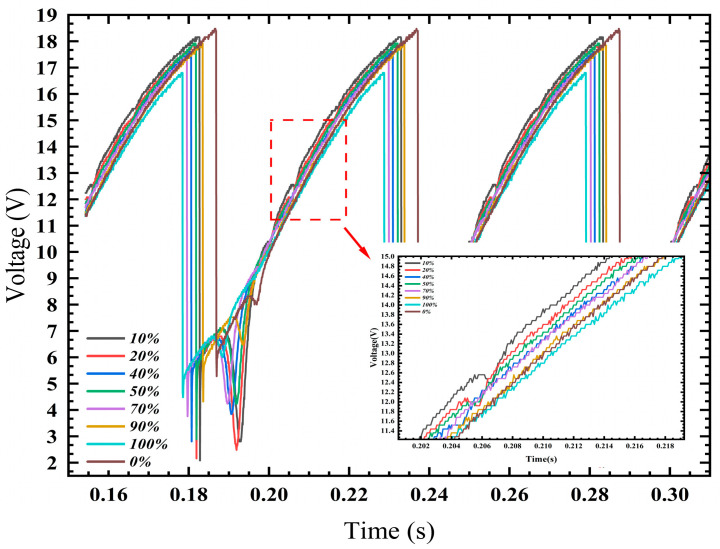
Absorption profiles of ethanol at different concentrations.

Following heating and evaporation, these were introduced into the gas absorption cell as mixed water vapor-ethanol samples, and their transmission spectral contours were acquired under identical experimental conditions. The results are presented in Figure 7.

### 3.4. Model Design and Experiments

#### 3.4.1. Dataset and Feature Extraction

A total of 1410 samples were collected in this study, covering ethanol gas at various concentrations as well as other gases (nitrogen and water vapor). Each sample was exported as a CSV file from a Tektronix TBS2204B oscilloscope, containing 2000 sampling points with a sampling interval of 8 × 10^−5^ s. All signals were manually inspected, and clearly anomalous contours caused by operational errors during the experiments were excluded.

To preserve the structural information of the spectral contours and avoid overfitting induced by high-dimensional features, uniform downsampling was employed to compress each sample from 2000 to 200 points.

Data preprocessing was then performed on each downsampled sample. First, baseline correction was applied: the minimum value of each sample was subtracted from all its data points to eliminate the DC offset caused by overall attenuation in the optical path. Subsequently, internal normalization was conducted: Z-score standardization was applied to the 200 corrected points by subtracting the mean and dividing by the standard deviation, yielding a waveform with a mean of 0 and a standard deviation of 1 for each sample [35]. This operation eliminates amplitude discrepancies arising from laser power fluctuations across different samples while preserving the relative shape information of the contours.

Ultimately, each sample was transformed into a 200-dimensional feature vector, where each component represents a normalized light intensity value. This feature vector is directly used as the input for the subsequent LightGBM model, without employing any handcrafted statistical features.

In terms of label definition, the prediction target for the regression task is the true concentration of ethanol vapor in the gas absorption cell. The ground truth concentration is obtained as follows [36,37,38]:

The vapor concentration is calculated using the ideal gas law and Raoult’s law, with the calculated value serving as the ground truth. First, the mole fraction of ethanol in the liquid phase, X_EtOH_, is calculated based on the volume fraction ϕ of the ethanol solution injected into the gas generation cell. Next, the Antoine equation is used to determine the saturated vapor pressure of pure ethanol, p_sat_, at the laboratory temperature T. Assuming phase equilibrium is achieved for the vapor above the liquid surface, the partial pressure of ethanol is given by p_EtOH_ = X_EtOH_ × p_sat_. The total pressure P_total_ inside the absorption cell is equal to the ambient atmospheric pressure; therefore, the volume concentration of ethanol vapor is expressed as:

(7) $$C_{\mathrm{ppm}}=\frac{p_{\mathrm{EtOH}}}{P_{\mathrm{total}}}\times 10^{6}$$

The experiments were conducted under a standard atmospheric pressure of 101.325 kPa, with evaporation carried out at a constant temperature of 26 °C and a fixed carrier gas flow rate of 200 mL/min. For non-ethanol samples, such as nitrogen and water vapor, the concentration label was set to 0 ppmv. The calculated vapor concentrations for ethanol solutions ranging from 5% to 100% (*v*/*v*) were derived based on Raoult’s law, covering values from 1263 ppmv (5%) to 78,950 ppmv (100%), as detailed in Table 1.

The dataset was randomly divided into training and test sets in an 8:2 ratio. The calculated vapor concentrations based on Raoult’s law were directly assigned as the regression labels. For the binary classification task, the labels were defined as “ethanol” (1) and “non-ethanol” (0).

It is important to acknowledge a potential bias in the theoretical concentration calculation. The concentrations derived from Raoult’s Law and the ideal gas law represent the theoretical maximum (saturation) concentrations under strict thermodynamic equilibrium. However, in our experimental setup, the vapor generation cell is continuously purged by carrier gas at a fixed flow rate of 200 mL/min to simulate dynamic leakage scenarios and ensure rapid response. This continuous purging prevents the headspace from reaching complete static saturation, meaning the actual ethanol vapor concentration is likely lower than the theoretical values calculated.

Nevertheless, this overestimation bias does not compromise the core objectives and conclusions of our work for the following reasons: (1) Dynamic Equilibrium and Repeatability: By strictly controlling the carrier gas flow rate and temperature, the system reaches a highly repeatable dynamic steady state. Although the absolute concentration is lower than the theoretical saturation value, the generated vapor concentration remains stable and highly reproducible for each specific liquid sample. (2) Monotonic Correlation for Machine Learning: The primary goal of this study is leakage detection and relative severity estimation, rather than absolute metrological quantification. The actual vapor concentration maintains a strict monotonically positive correlation with the theoretical concentration. Machine learning models, such as the proposed LightGBM, are inherently capable of learning these robust relative mappings and nonlinear trends from the spectral features. Therefore, the algorithm’s performance is largely insensitive to a systematic global offset in the absolute concentration labels. While obtaining absolute ground-truth validation using a calibrated ethanol vapor generator or a high-precision reference sensor (e.g., Gas Chromatography-Mass Spectrometry) would be ideal for metrological traceability, it falls outside the current scope of this engineering-focused study.

#### 3.4.2. Hyperparameter Settings

To ensure optimal model performance and provide a transparent justification for the selected hyperparameters, we performed a systematic hyperparameter optimization based on the expanded dataset of 1410 samples. A two-stage strategy was adopted [39]:

In the first stage, a randomized search with 200 iterations and 3-fold cross-validation was employed to explore a broad parameter space. Five key hyperparameters were included in the search: num_leaves, learning_rate, feature_fraction, min_child_samples, and reg_lambda. The cross-validation AUC was used as the optimization criterion. This coarse search efficiently identified the promising parameter region.

In the second stage, a refined grid search with 5-fold cross-validation [31,39] was conducted around the optimal region discovered in the first stage to precisely determine the final configuration. The optimized hyperparameters are listed in Table 2.

**Figure 8:**
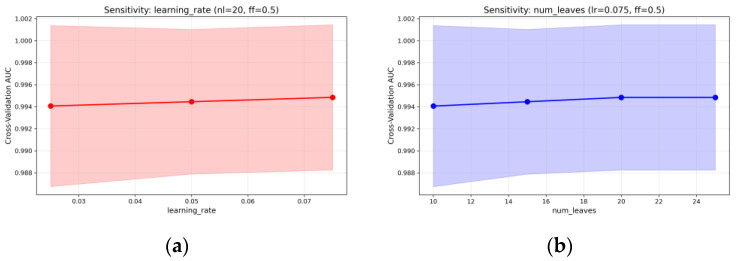
Sensitivity analysis of LightGBM hyperparameters: (**a**) AUC variation with learning_rate; (**b**) AUC variation with num_leaves.

To evaluate the robustness of the model with respect to hyperparameter variations, we conducted a sensitivity analysis by varying num_leaves and learning_rate while keeping the other parameters fixed at their optimal values. As shown in Figure 8, the model maintains stable performance (AUC fluctuation < 0.005) over a wide range of num_leaves (10–30) and learning_rate (0.04–0.12). This stability is particularly advantageous for industrial deployment, where minor parameter variations may occur due to software environment updates or hardware differences.

#### 3.4.3. Model Evaluation

To comprehensively evaluate the performance of the LightGBM model based on raw waveform inputs in the tasks of ethanol concentration prediction and leakage detection, this study designed two types of experiments: regression and classification. All experiments employed 5-fold cross-validation to avoid the randomness introduced by a single data split, and all metrics are reported in the format of “mean ± standard deviation”.

To avoid data leakage, the dataset was split at the concentration-group level rather than at the individual sample level. Specifically, all repeated measurements corresponding to the same ethanol concentration were grouped together, and the split into training and test sets was performed on these concentration groups using stratified grouping to ensure that samples from the same concentration did not appear in both the training and test sets simultaneously. This approach prevents the model from learning batch-specific noise or experiment-specific artifacts, thereby providing a more honest estimate of its generalization performance.

To assess the repeatability and reproducibility of the proposed system, multiple measurements were conducted under both identical and varied conditions. Repeatability was evaluated by collecting repeated spectral measurements at each concentration level under the same experimental conditions.

To demonstrate the advantages of LightGBM, the following three mainstream methods were selected for comparison:

PLS Regression [26]: Partial Least Squares regression, where the number of principal components is determined via cross-validation. It takes the same 200-dimensional waveform features as LightGBM as input, with feature standardization applied.

Random Forest [19]: Comprising 100 decision trees with a maximum depth of 10, directly utilizing the raw waveform features.

Peak Method: To provide a comprehensive baseline comparison, the traditional Peak Absorption Method (hereafter referred to as the Peak Method) was also implemented and evaluated. Unlike multivariate approaches that utilize full-band or adjacent-band spectral information, the Peak Method is a univariate approach that directly relies on the absorbance value at the characteristic peak wavelength of ethanol.

MLP [40]: MLP is a fully connected feedforward neural network consisting of an input layer, multiple hidden layers with nonlinear activation functions, and an output layer. It serves as a baseline deep learning model to evaluate whether simple nonlinear transformations of the handcrafted features are sufficient for the detection task.

1D-CNN [41]: 1D-CNN applies convolutional kernels along the spectral dimension to automatically extract local features from adjacent wavelength points. This architecture is particularly suitable for spectral data, as it can capture localized patterns such as peak shapes and slopes while preserving spatial locality.

LSTM [28]: LSTM is a type of recurrent neural network designed to model sequential dependencies by selectively retaining or forgetting information over long sequences. It is well-suited for spectral contour analysis, where the order of wavelength points carries contextual information that may be relevant for concentration prediction.

CNN-LSTM [42]: CNN-LSTM combines the strengths of both architectures: the CNN layers first extract local spectral features from the input contour, and the LSTM layers then model the sequential relationships among these features. This hybrid structure captures both local patterns and global dependencies, making it a powerful candidate for complex spectral analysis tasks.

##### Experimental Evaluation of the Binary Classification Task

To evaluate the binary classification performance, we compared LightGBM against Random Forest and Partial Least Squares regression using 5 × 2 cross-validation. As shown in Table 3, LightGBM achieved the highest mean AUC of 0.9949 ± 0.0043, followed by Random Forest with 0.9852 ± 0.0046, while PLS performed poorly with a mean AUC of only 0.7658 ± 0.1960. The extremely large standard deviation of PLS indicates that linear models are inherently inadequate for decoupling the severe spectral overlap between ethanol and water vapor.

Paired *t*-tests confirmed that the differences between LightGBM and both Random Forest and PLS were statistically significant (*p* = 0.0044, and PLS, *p* = 0.0078, were statistically significant.

We also selected several commonly used deep learning models for comparison, including MLP, 1D-CNN, LSTM, and CNN-LSTM. All deep learning models achieved lower AUC than LightGBM; the highest among them was LSTM with 0.9878, while exhibiting inference speeds 17 to 265 times slower and larger model footprints. These results confirm that deep learning offers no accuracy advantage for this specific task while incurring substantial penalties in computational efficiency.

From an engineering deployment perspective, inference speed is a critical factor for production line integration. LightGBM achieved an inference time of 0.0058 ms per sample under CPU single-thread conditions, which is 2.9 times faster than Random Forest at 0.0166 ms per sample. PLS exhibited the fastest inference at 0.0008 ms per sample, but its unacceptably low AUC renders it unsuitable for reliable leakage detection.

Feature importance analysis based on the Gain metric was conducted to reveal which spectral features contribute most to the model’s decision-making process. As shown in Table 4, the maximum intensity (Max) ranks first with a contribution of 46.2%, followed by the mean intensity (Mean) at 27.6%. Together, these two baseline-related features account for nearly three-quarters of the total importance, indicating that the model primarily relies on the non-absorbed reference level of the waveform to establish a baseline for ethanol detection.

The minimum intensity (Min), which directly corresponds to the absorption valley, contributes only 4.8%, suggesting that the model does not simply read the valley depth in isolation but instead combines baseline and valley information—a strategy consistent with the principle of differential absorption spectroscopy. The ArgMax (peak position) contributes 8.3%, implying that waveform morphology helps the model correct for background drift caused by environmental fluctuations. In contrast, the ArgMin (valley position) contributes merely 0.5%, confirming that the ethanol absorption peak remains stable at approximately 1392 nm with negligible wavelength shift. The remaining features play secondary roles. These results demonstrate that the model has automatically learned a physically meaningful strategy—establishing a baseline via Max and Mean, detecting the absorption dip via Min, and correcting drift via ArgMax—which explains its strong robustness against water vapor interference and enhances its credibility for industrial deployment.

Overall, LightGBM outperformed all competing models across two dimensions critical for industrial deployment: classification accuracy with an AUC of 0.9949 and inference speed of 0.0058 ms per sample.

##### Experimental Evaluation of the Regression Task

We compared LightGBM against Random Forest, Partial Least Squares regression, and four deep learning models (MLP, 1D-CNN, LSTM, CNN-LSTM) using 5-fold cross-validation for the regression task. As summarized in Table 5, LightGBM achieved the best performance with an R^2^ of 0.5854 ± 0.0919, outperforming Random Forest (0.4968 ± 0.0930), PLS (0.2420 ± 0.0696), and all deep learning models. Among the deep learning models, 1D-CNN achieved the highest R^2^ of 0.5509 ± 0.0681, slightly below LightGBM, followed by MLP with 0.4915 ± 0.0572.

However, both LSTM and CNN-LSTM produced negative R^2^ values of −0.8316 ± 0.0703 and −0.8322 ± 0.0703, respectively, indicating that these recurrent architectures severely overfit the regression task. This is likely due to the limited sample size of 1410, which is insufficient for training sequence models with a large number of parameters. It is worth noting that while the LSTM performed reasonably well in the binary classification task (AUC = 0.9878), its regression performance deteriorated drastically, suggesting that classification and regression tasks have fundamentally different requirements: classification benefits from the ability to capture global waveform patterns, whereas regression demands precise quantitative differentiation of subtle concentration differences, which is considerably more challenging with the current dataset size.

The corresponding MAE and RMSE for LightGBM were 7563.2 ± 1932.2 ppmv and 25,700.9 ± 5328.0 ppmv, respectively. The poor performance of PLS and the LSTM-based models confirms that neither linear models nor complex recurrent architectures are capable of reliably capturing the nonlinear relationship between spectral features and concentration with the current data scale, while tree-based ensemble methods like LightGBM offer a better balance of accuracy and generalization.

From an engineering perspective, the regression model serves as a complementary tool for assessing leakage severity. Although the absolute concentration prediction is affected by the lack of gas chromatography calibration, the model reliably ranks samples by their ethanol concentration levels, enabling the system to differentiate between minor and severe leaks. This information assists production line operators in prioritizing bottles for further inspection or rejection.

We also analyzed the feature importance of the LightGBM regression model based on the Gain metric to identify which spectral features contribute most to concentration prediction. As shown in Table 6, the ranking differs notably from the classification task. The minimum intensity (Min) ranks first with a contribution of 28.7%, followed by the mean intensity (Mean) at 22.4% and the maximum intensity (Max) at 18.9%.

The dominance of Min in the regression task indicates that the absorption valley depth is the most critical factor for quantitative concentration prediction, which aligns with the Beer–Lambert law, where absorption intensity is directly proportional to concentration. In contrast, the classification task relied more heavily on baseline features (Max and Mean), as distinguishing ethanol from non-ethanol primarily required identifying the presence of an absorption dip rather than measuring its precise depth. The ArgMin (valley position) contributes 9.2%, suggesting that the exact location of the absorption valley provides useful information for concentration quantification, possibly due to slight peak shifts caused by varying ethanol-water vapor mixtures. The ArgMax (peak position) contributes 6.8%, indicating that waveform morphology and baseline drift also play a role in concentration prediction, though less prominently than in classification. The remaining features—Std (6.2%), Median (5.1%), and Range (2.7%)—provide supplementary information.

These results demonstrate that the regression model focuses more on the absorption depth itself, while the classification model emphasizes baseline-reference comparison, reflecting the fundamentally different requirements of the two tasks: regression demands precise measurement of the absorption magnitude, whereas classification only needs to detect the presence of absorption features.

#### 3.4.4. Model Performance Evaluation Under Interference Conditions

As shown in Table 7, to evaluate the anti-interference capability of the model, we constructed a dedicated test set consisting of 422 samples from four different sources. Specifically, we randomly selected 120 samples from the original dataset, collected 50 samples under varying carrier gas flow rates, acquired 52 samples at different ambient temperatures, and purchased four bottles of commercially available wines with different concentrations and types, from which 200 samples were collected. These 422 samples were then used as the test set for the model anti-interference experiment.

As shown in Table 8, for the classification task, the model achieved an overall accuracy of 0.94, with an alcohol recall of 0.99 and a non-alcohol precision of 0.99. This means that 99% of alcoholic samples were correctly detected, corresponding to a false negative rate of only 1%, while 92% of non-alcoholic samples were correctly classified, with a false positive rate of approximately 8%. The alcohol precision reached 0.88, indicating that 88% of the samples identified as alcohol were indeed positive. These results confirm that the model maintains near-perfect sensitivity for detecting leaks while keeping false alarms at a manageable level.

For the regression task, which predicts the continuous ethanol vapor concentration, the model achieved a coefficient of determination (R^2^) of 0.8651 ± 0.0950, with a mean absolute error of 2813.3 ± 343.5 ppmv and a root mean square error of 7098.2 ± 2027.7 ppmv. The relative prediction error, defined as MAE divided by the full concentration range (78,950 ppmv), is approximately 3.6%, confirming the model’s capability for accurate quantitative concentration prediction even under varying environmental conditions.

Together, these results demonstrate that the LightGBM model not only reliably detects the presence of ethanol leakage (classification) but also provides accurate estimation of leakage severity (regression) under diverse and challenging conditions. The consistency between classification and regression performance on the same anti-interference test set strongly validates the model’s robustness and its readiness for real-world industrial deployment.

#### 3.4.5. Testing and Application

**Figure 9:**
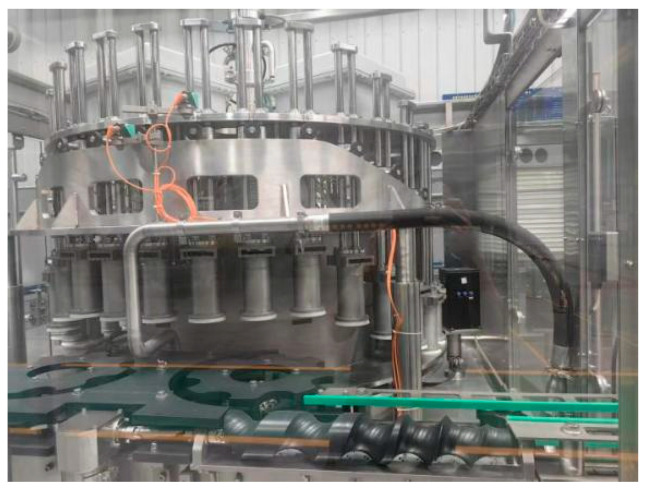
Schematic diagram of the actual production line test.

The ultimate goal of this study is to develop a leakage detection system applicable to real production lines as shown in Figure 9, with particular emphasis on identifying micro-leakages that are difficult to detect through conventional visual or weight-based inspection methods.

To this end, we designed a gas extraction scheme based on negative-pressure sampling: a custom enclosure is placed over each bottle, and a suction pump draws the headspace gas into an absorption cell for spectral analysis, thereby accelerating the vaporization of any leaked liquid and enabling rapid detection even for extremely small amounts of leakage.

**Figure 10:**
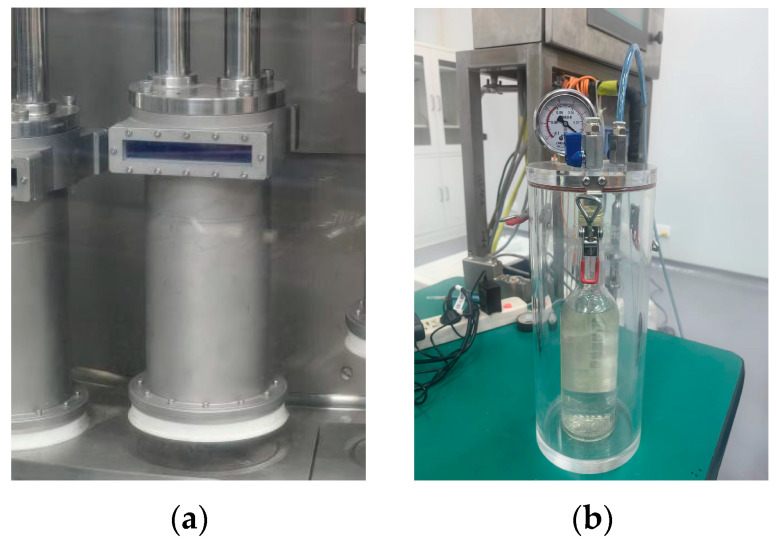
Custom-designed bottle enclosures: (**a**) Custom-designed metallic bottle enclosure on the actual production line; (**b**) Acrylic bottle enclosure of the same volume for laboratory testing.

Since the actual production line was still under construction and direct online deployment was not feasible at the time of this study, we custom-built an acrylic enclosure with the same volume as the metallic enclosure shown in Figure 10a to validate the proposed scheme under simulated conditions. Figure 10b shows the acrylic enclosure used for laboratory testing.

Four types of commercially available wines were purchased, and tiny artificial defects—such as micro-cracks and pinholes—were introduced into the bottles to realistically simulate micro-leakage scenarios. The defective bottles were placed into the chamber, and the internal pressure was reduced to 0.04 MPa via a vacuum pump, consistent with the negative-pressure principle of the production line design. This negative-pressure environment accelerates the vaporization of trace amounts of leaked liquid, effectively amplifying the spectral signal and making it detectable. Spectral data were collected from these simulated micro-leakage scenarios, yielding a total of 320 samples.

The experimental results on these 320 simulated micro-leakage samples are summarized in Table 9. The LightGBM model achieved an overall accuracy of 0.95, with an alcohol recall of 1.00, meaning that all 260 alcoholic samples were correctly detected with zero false negatives. The alcohol precision reached 0.95, indicating that 95% of the samples identified as alcohol were indeed positive. The non-alcohol recall was 0.75, corresponding to a false positive rate of 25%. The AUC score was approximately 0.88, reflecting strong overall discriminative ability.

These results confirm that the proposed negative-pressure extraction scheme, combined with the LightGBM model, effectively captures subtle leakage signals even under simulated micro-leakage conditions using real commercial wine samples. The zero false negatives achieved in this experiment are particularly noteworthy, as missed leaks are the most critical failure mode in industrial leakage detection—any missed leak could result in defective products reaching consumers, causing potential safety hazards and brand reputation damage.

Building upon these validated laboratory results, we further implemented the gas extraction scheme on an actual liquor bottle packaging and filling line. The specific procedure is as follows: first, the test bottle is placed into a custom-designed bottle enclosure as shown in Figure 10a, and background sample data are collected at the 0-s mark. At the 3-s mark, the suction pump is activated to slowly draw the gas from the enclosure into the coupled gas absorption cell while data collection begins, thereby maintaining a negative pressure inside the enclosure. If the bottle is leaking, substances such as ethanol and water inside will undergo rapid vaporization; conversely, if the bottle is intact, no such changes will occur within the enclosure. Once the pressure inside the enclosure reaches −60 kPa, the suction pump is turned off, and the collected data are processed.

Subsequently, the software automatically invokes the pre-trained LightGBM classification model to determine the presence of ethanol and the regression model to calculate the predicted ethanol concentration for the bottle based on the extracted time-domain features, compares it with the background concentration, and outputs the concentration increment. If the increment exceeds the preset threshold, the system triggers an audible and visual alarm, identifies the bottle as having a leakage risk, and automatically diverts it to the re-inspection lane. Conversely, if the increment is within the threshold, the bottle is deemed well-sealed and allowed to proceed to the next process. The entire detection process takes no more than 5 s per bottle, enabling non-destructive, rapid, and online determination of packaging leakage.

## 4. Conclusions

This paper presents a machine learning-based near-infrared laser leakage detection system for wine bottles, addressing the limitations of conventional visual, weight-based, and acoustic inspection methods for detecting micro-leakages. The main conclusions and contributions are summarized as follows:

A 1392 nm tunable diode laser was selected as the detection light source based on the characteristic absorption peak of ethanol vapor in the near-infrared region. Differential spectroscopy experiments clearly revealed the spectral distinctions between ethanol and water vapor, providing a physical foundation for subsequent machine learning-based classification and regression. The complete experimental platform, including the gas generation system, the 3-m optical path absorption cell, and the laser control and signal acquisition hardware, was successfully constructed and validated.

In terms of data processing, each raw 2000-point spectral contour was compressed into a 200-dimensional feature vector through baseline correction, Z-score normalization, and uniform downsampling. A systematic two-stage hyperparameter optimization strategy combining randomized search and refined grid search yielded the optimal LightGBM configuration with a 5-fold cross-validation AUC of 0.9949. Sensitivity analysis confirmed that the model maintains stable performance over wide parameter ranges, demonstrating its robustness for industrial deployment.

For the binary classification task, LightGBM achieved an AUC of 0.9949 and an inference speed of 0.0058 ms per sample on a CPU, outperforming Random Forest, PLS, and four representative deep learning models. Feature importance analysis revealed that the model primarily relies on baseline intensity features combined with the absorption valley to determine the presence of ethanol, a strategy consistent with the principle of differential absorption spectroscopy. For the regression task, LightGBM achieved an R^2^ of 0.8651 ± 0.0950, an MAE of 2813.3 ± 343.5 ppmv, and an RMSE of 7098.2 ± 2027.7 ppmv on the anti-interference test set. The importance ranking in regression shifted notably, with the minimum intensity becoming the most critical feature, aligning with the Beer–Lambert law, where absorption depth is directly proportional to concentration.

The anti-interference experiment on 422 samples under varying flow rates, temperatures, and commercial wine types confirmed the model’s robustness, achieving an overall accuracy of 0.94, an AUC of 0.97, and an alcohol recall of 0.99. Furthermore, a simulated micro-leakage test using 320 samples from artificially damaged commercial wine bottles in a custom-built acrylic vacuum chamber achieved an accuracy of 0.95 with zero false negatives, validating the effectiveness of the proposed gas extraction scheme in capturing subtle leakage signals. The complete detection cycle takes no more than 5 s per bottle, enabling non-destructive, rapid, and online packaging integrity assessment.

Despite these encouraging results, several limitations should be acknowledged. The ethanol vapor concentrations used for regression training were derived from theoretical calculations rather than direct measurement, due to equipment cost constraints. Actual concentrations are likely lower than the theoretical values owing to continuous nitrogen purging. In addition, production-line validation was not yet conducted as the line was still under construction.

Future work will focus on several directions. First, validation using a calibrated gas chromatograph or a high-precision ethanol vapor generator will be pursued to obtain absolute concentration ground truth. Second, online deployment and testing on the actual production line will be carried out once the line becomes operational. Third, the training dataset will be expanded to include a broader range of interferers to further enhance the model’s selectivity and robustness in complex industrial environments. Finally, model compression and deployment on embedded devices will be explored to facilitate wider adoption on high-speed packaging lines.

## Figures and Tables

**Table 1:** Summary of Datasets with Different Concentrations.

Ethanol Fraction (%)	5	10	15	20	25	30	35	40	50	60	70	80	90	100	0
Theoretical Concentration (ppmv)	1263	2621	4082	5660	7366	9230	11,250	13,480	18,620	24,990	33,060	43,630	58,070	78,950	0
Number of Samples	82	82	82	82	83	82	40	82	103	83	61	61	61	83	402

**Table 2:** Model experimental hyperparameters.

Parameter	Value
num_leaves	20
learning_rate	0.075
num_boost_rounds	300
feature_fraction	0.5
bagging_fraction	0.8
bagging_freq	5
reg_lambda	0.1
min_child_samples	15
early_stopping_rounds	20

**Table 3:** Comparison of binary classification models.

Model	AUC	Inference Time (ms/Sample)
LightGBM	0.9949 ± 0.0043	0.0058
Random Forest	0.9852 ± 0.0046	0.0166
PLS	0.7658 ± 0.1960	0.0008
MLP	0.9563 ± 0.0630	0.10 ± 0.01
1D-CNN	0.9837 ± 0.0267	0.67 ± 0.09
LSTM	0.9878 ± 0.0148	1.54 ± 0.10
CNN-LSTM	0.9848 ± 0.0160	0.92 ± 0.02

**Table 4:** Feature importance ranking of the binary classification model.

Rank	Feature	Gain Contribution (%)
1	Max	46.2
2	Mean	27.6
3	ArgMax	8.3
4	Std	6.0
5	Median	5.3
6	Min	4.8
7	Range	1.4
8	ArgMin	0.5

**Table 5:** Comparison of regression models.

Model	MAE (ppmv)	RMSE (ppmv)	R^2^
LightGBM	7563.2 ± 1932.2	25,700.9 ± 5328.0	0.5854 ± 0.0919
Random Forest	8864.6 ± 1694.1	28,353.1 ± 5573.2	0.4968 ± 0.0930
PLS	18,539.0 ± 2476.2	34,989.5 ± 6342.0	0.2420 ± 0.0696
MLP	10,870.6 ± 501.1	16,564.7 ± 778.3	0.4915 ± 0.0572
1D-CNN	10,300.5 ± 730.0	15,542.6 ± 1003.1	0.5509 ± 0.0681
LSTM	21,286.5 ± 1375.5	31,527.8 ± 1515.7	−0.8316 ± 0.0703
CNN-LSTM	21,292.8 ± 1375.7	31,533.3 ± 1515.8	−0.8322 ± 0.0703

**Table 6:** Feature importance ranking of the regression model.

Rank	Feature	Gain Contribution (%)
1	Min	28.7
2	Mean	22.4
3	Max	18.9
4	ArgMin	9.2
5	ArgMax	6.8
6	Std	6.2
7	Median	5.1
8	Range	2.7

**Table 7:** Composition of the anti-interference test set.

Data Source	Number of Samples	Condition Description
Original dataset (randomly selected)	120	Randomly sampled from the original 1410 samples
Variable carrier gas flow rate	50	Collected under different flow rates
Variable ambient temperature	52	Collected at different room temperatures
Commercial wines	200	Four bottles of commercially available wines with different concentrations and types
Total	422	—

**Table 8:** Anti-interference test results.

Task	Metric	Value
Classification	Accuracy	0.94
	AUC	0.97
	Alcohol Recall	0.99
	Alcohol Precision	0.88
	Non-alcoholic recall	0.92
Regression	R^2^	0.8651 ± 0.0950
	MAE	2813.3 ± 343.5 ppmv
	RMSE	7098.2 ± 2027.7 ppmv

**Table 9:** Results of the simulated production line test.

Class	Precision	Recall	F1-Score	Support
Non-alcoholic	1.00	0.75	0.86	60
Alcohol	0.95	1.00	0.97	260
Accuracy	-	-	0.95	320
Macro Avg	0.98	0.88	0.92	320
Weighted Avg	0.96	0.95	0.95	320
AUC	0.88			

## Data Availability

Data will be made available on request.
